# Robotic-assisted surgery for endometrial cancer: a comparison of surgical and oncologic outcomes in patients with low and high BMI at an Indian tertiary care center

**DOI:** 10.1007/s11701-023-01747-z

**Published:** 2024-01-11

**Authors:** Rama Joshi, Rashmi Rekha Bora, Tarini Sonwani

**Affiliations:** Department of Gynae Oncology and Robotic Surgery, Fortis Memorial Research Institute (FMRI), Sector-44, Opposite HUDA City Centre, Gurugram, Haryana 122002 India

**Keywords:** Endometrial cancer, Robotic-assisted surgery, Obese patients, Morbidly obese patients, BMI

## Abstract

The robotic-assisted surgery for endometrial cancer (EC) is becoming increasingly important, owing to the superior surgical outcomes. However, efficacy data from India is limited, particularly for older women who are obese. We undertook this study to compare the surgical outcomes of robotic-assisted surgery among Indian EC patients with a BMI of < 30 and ≥ 30 kg/m^2^. A retrospective chart review was conducted for the period of May 2016 to October 2020. Data on patient demographics, medical history, clinical characteristics, and perioperative outcomes were collected by a single senior surgeon, followed by statistical analysis. A total of 99 patients; 39 in the BMI group < 30 and 60 in the BMI group ≥ 30 kg/m^2^ were included in the study. The mean age of the BMI groups < 30 and ≥ 30 kg/m^2^ was 60.92 ± 10.43 and 58.90 ± 8.52 years respectively (P = 0.2944). The mean total operating time was slightly higher in the BMI group < 30 kg/m^2^ (P = 0.8552) but the difference was not statistically significant. Similarly, the mean blood loss (P = 0.2041), length of hospital stays (P = 0.6564), early (P = 0.7758) and delayed complications (P = 0.1878) were less in the BMI group < 30 kg/m^2^ but the difference was not statistically significant either. At a median follow-up of 22.3 months, the number of recurrences (5.13% vs 3.33%) and deaths (2.56% vs 1.67%) were more in BMI < 30 kg/m^2^ group. Our study suggests that obese older women predisposed to multiple medical co-morbidities and surgical complications would especially benefit from robotic-assisted technology regardless of their BMI.

## Introduction

Endometrial cancer (EC), also known as uterine corpus cancer, accounts for about 4.5% of the global incidence of cancer and 2.2% of cancer deaths in women [1]. In 2020, nearly 0.42 million women around the world were diagnosed with EC [1], and cases are expected to increase exponentially over the next decade. In India, the EC ranks third among women following cancer of the cervix and ovary. While 26,514 new cases were observed in 2020, the EC is expected to represent 3.7% of all female cancer cases by 2025 [2].

Although a causative association between obesity and gynecology cancers is not entirely understood, an increased risk for EC with increased Body Mass Index (BMI) is well documented due to perimenopausal hormonal changes [3–5]. A five-unit increase in BMI can result in a 50% increase in the risk of EC [5]. Moreover, the relative risk of disease-specific mortality increases from 2.53 for obese women (BMI 30–34.9 kg/m^2^) to 6.25 for morbidly obese (BMI > 40 kg/m^2^) women [6]. In a retrospective study, morbidly obese, early EC patients had higher mortality rates than non-obese women [7]. It has been reported, that 1 in 35 women has a lifetime risk of developing EC which increases steadily with age [8]. Nevertheless, a young population cannot be excluded where 14% of the cases are observed in premenopausal women and 5% in women under 40 years of age*.* The 5-year overall survival (OS) is 90% for the early-stage EC but is reduced significantly to 60% and 20% for stages III and IV respectively [3]. The challenge increases significantly in obese elderly patients, who have a higher overall risk of dying [9].

Surgery with or without adjuvant treatment is the current gold standard in 80% of cases of early-stage, uterine-confined, or FIGO (International Federation of Gynaecology and Obstetrics) stage I disease [10]. Conventionally, a total abdominal hysterectomy and a staged bilateral salpingo-oophorectomy were performed with open laparotomy, which can result in prolonged hospital stays and postoperative complications due to its invasiveness. Over the last 20 years, minimally invasive laparoscopic surgery has replaced the former, but problems related to blood loss, longer operation time, and hospital stay as well as steep learning curve have not been eliminated. The benefit is also debatable for people with BMI > 40 kg/m^2^ and weak elderly patients [11, 12].

More recently a minimally invasive robotic-assisted surgical approach has been widely adopted, particularly in obese and high-risk patients due to smaller incisions and less post-operative complications [13]. The robotic-assisted approach provides 7 degrees of movement with a fatigue-resistant robot hand, improved and stable 3D visualization, and a faster learning curve that complements the comfort and precision of surgeons. Many studies have shown a decrease in blood loss, a reduction in operating time, higher lymph node yield, and improved mortality through the robotic-assisted technique as compared to both laparoscopy and laparotomy. As the torque experienced by the abdominal wall is lower, overall pain and tissue trauma are reduced leading to an early return to productive life [14].

At present, the treatment of EC using a robotic-assisted approach is still naïve in India. As a result, data on efficacy are scarce, particularly in elderly women who are obese. The purpose of this study is to compare the results of robotic-assisted surgery in Indian EC patients with a BMI of < 30 kg/m^2^ and ≥ 30 kg/m^2^.

## Methods

### Study design, surgical technique, and efficacy endpoints

The study was conducted in the Department of Gynae Oncology and Robotic Surgery of the Fortis Memorial Research Institute, Gurugram. A retrospective chart review for the period of May 2016 to October 2020 was performed for overweight and obese patients who underwent minimally invasive robotic-assisted surgery for the treatment of EC. The main efficacy endpoints were to compare perioperative outcomes across subgroups of patients with a BMI of < 30 kg/m^2^ and ≥ 30 kg/m^2^. The Robotic surgery was performed using Da Vinci Si Surgical System (Intuitive Surgical, Sunnyvale, CA, USA). It consists of a 3D vision system and Endowrist instruments with 7 degrees of freedom to recreate dexterity and a range of movement for a high degree of precision and flexibility.

A single skilled surgeon performed all the procedures. All patients underwent complete staging surgery for uterine malignancy with hysterectomy, salpingo-oophorectomy, lymph node dissection (pelvic with/without para-aortic nodes), and omentectomy and peritoneal biopsies when required.

All patients received preoperative antibiotic prophylaxis and bowel preparation. Six ports were inserted through the anterior abdominal wall after creating pneumoperitoneum using a standard technique with a Veress needle. A 12 mm camera port was placed 3 cm above the umbilicus in the midline. Two 8-mm ports were placed, one on the right side for monopolar curved scissors and the other one on the left for bipolar. One 8-mm instrument port was placed in the left upper quadrant 5 cm above the anterior superior iliac spine for the ProGrasp. A 12-mm assistant port was placed 5 cm above the anterior superior iliac spine in the right upper quadrant and a 5-mm suction port was placed on the right side. The patient was placed in a steep Trendelenburg position at 29°. Docking of the Si system was done at the ports. Peritoneal washings were taken through the suction port, followed by staging surgery by the primary surgeon at the Da Vinci Si console and the assistant surgeon at the right of the patient. Specimens were retrieved vaginally at the end of the procedures.

### Data collection and ethical considerations

Demographic data, medical history, clinical characteristics, and perioperative outcomes were collected from the patient’s medical records. Data points recorded were Age, BMI, parity, co-morbidities, uterine size, stage, histopathological findings, number of nodes harvested, operative time, intraoperative blood loss, length of hospital stays, early and delayed post-operative complications, adjuvant treatment, disease-free survival (DFS) and OS. The Clavien–Dindo classification was used to grade complications. The study was carried out in accordance with the ethical principles set out in the latest version of the Declaration of Helsinki, and the applicable guidelines for good clinical practice. Ethics committee approval was taken for the study vide letter number 2020-006-TH-28.

### Statistical analysis

The statistical analysis of the continuous variables was summarized by the arithmetic mean, and standard deviation (SD). An independent sample t-test was used to compare the mean values of parameters such as age, uterine size, number of nodes, and operative outcomes across the study groups. Categorical variables were summarized using frequencies and percentages. Statistical analysis was performed using Pearson’s Chi-square to check the association between the variables. A two-sided P < 0.05 was considered significant. DFS and OS for the study groups were analyzed using the Kaplan–Meier analysis. Statistical analysis was performed using SAS version 9.4 (SAS Institute Inc., North Carolina, USA).

## Results

### Baseline characteristics of participants

A total of 99 patients with an average age of 59.7 ± 9.37 years were included in the study. There were 39 patients in the BMI group < 30 kg/m^2^ and 60 patients in the BMI group ≥ 30 kg/m^2^. The baseline characteristics of study participants are shown in Table 1.

**Table 1:** Demographic data and medical history

Demographics data and medical history	All (N = 99)	BMI < 30 kg/m^2^ (N = 39)	BMI ≥ 30 kg/m^2^ (N=60)	P value
Age, mean ± SD, year	59.70 ± 9.37	60.92 ± 10.43	58.90 ± 8.52	0.2944*
BMI, mean ± SD, kg/m^2^	32.37 ± 6.21	27.30 ± 1.96	35.67 ± 5.79	<0.0001*
Parity, n (%)				
0	8 (8.08)	5 (12.82)	3 (5.00)	0.2583^#^
1	14 (14.14)	2 (5.13)	12 (20.00)	
2	46 (46.46)	18 (46.15)	28 (46.67)	
3	22 (22.22)	9 (23.08)	13 (21.67)	
4	7 (7.07)	4 (10.26)	3 (5.00)	
5	2 (2.02)	1 (2.56)	1 (1.67)	
Co-morbidities, n (%)				
Hypertension	59 (59.60)	22 (56.41)	37 (61.67)	0.4444^#^
Diabetes	28 (28.28)	11 (28.21)	17 (28.33)	
HTHY	24 (24.24)	8 (20.51)	16 (26.67)	
Hypothyroid	18 (18.18)	9 (23.08)	9 (15.00)	
Asthma	7 (7.07)	4 (10.26)	3 (5.00)	
Others	31 (31.31)	8 (20.51)	23 (38.33)	
Family history				
Yes	15 (15.15)	8 (20.51)	7 (11.67)	0.2327^#^
No	84 (84.85)	31 (79.49)	53 (88.33)	
Previous surgery				
Yes	64 (64.65)	24 (61.54)	40 (66.67)	0.6038^#^
No	35 (35.35)	15 (38.46)	20 (33.33)	
Previous surgery type				
Gynaecology/obstetrics	43 (43.43)	17 (43.59)	26 (43.33)	0.8907^#^
Abdominal	15 (15.15)	5 (12.82)	10 (16.67)	
Other	6 (6.06)	2 (5.13)	4 (6.67)	

Except for BMI, there were no significant differences in baseline characteristics across the study groups. Compared with the < 30 kg/m^2^ group, the mean BMI was significantly higher in the ≥ 30 kg/m^2^ group (27.30 ± 1.96 vs. 35.67 ± 5.79 kg/m^2^, P < 0.0001)

*Student’s t-test

^#^Chi-squared test

### Operative outcomes

A comparison of surgical outcomes between both groups is presented in Table 2.

**Table 2:** Operative outcomes

Operative outcomes	All (N = 99)	BMI < 30 kg/m^2^ (N = 39)	BMI ≥ 30 kg/m^2^ (N = 60)	P value
Blood loss, mean ± SD, ml	14.85 ± 13.15	12.76 ± 8.64	16.19 ± 15.22	0.2041*
Operative time, mean ± SD, min	282.77 ± 69.95	284.36 ± 80.85	281.73 ± 61.83	0.8552*
Length of hospital stay, mean ± SD, days	1.65 ± 0.65	1.62 ± 0.49	1.68 ± 0.74	0.6564*

*Student’s t-test

The mean total operating time was slightly higher in the BMI group < 30 kg/m^2^ but the difference was not statistically significant (P = 0.8552). Similarly, the mean blood loss (12.76 ± 8.64 vs. 16.19 ± 15.22 ml, P = 0.2041) and length of hospital stay (1.62 ± 0.49 vs. 1.68 ± 0.74, days, P = 0.6564) were lower in the BMI group < 30 kg/m^2^ but the difference was not statistically significant.

### Post-operative complications

Early and delayed post-operative complications are presented in Tables 3 and 4. Mean early (6 vs. 8, P = 0.7758) and delayed complications (2 vs. 8, P = 0.1878) were fewer in the BMI group < 30 kg/m^2^ but the difference was not statistically significant (Table 3).

**Table 3:** Post-operative complications (early and delayed)

Post-operative complications	All (N = 99)	BMI < 30 kg/m^2^ (N = 39)	BMI ≥ 30 kg/m^2^ (N = 60)	P value
Early complications, n (%)	14 (14.14)	6 (15.38)	8 (13.33)	0.7758^#^
Abdominal wound infection	1 (1.01)	0 (0.00)	1 (1.67)	
Anaemia	1 (1.01)	0 (0.00)	1 (1.67)	
Ileus	4 (4.04)	1 (2.56)	3 (5.00)	
Nausea	1 (1.01)	1 (2.56)	0 (0.00)	
Urinary tract infection (UTI)	5 (5.05)	2 (5.13)	3 (5.00)	
UTI and persistent low-grade pyrexia	1 (1.01)	1 (2.56)	0 (0.00)	
Wound gap, discharge	1 (1.01)	1 (2.56)	0 (0.00)	
Delayed complications, n (%)	10 (10.10)	2 (5.13)	8 (13.33)	0.1878^#^
Lymphocyst	2 (2.02)	0 (0.00)	2 (3.33)	
Parasthesias	2 (2.02)	0 (0.00)	2 (3.33)	
Pelvic lymphocyst	5 (5.05)	2 (5.13)	3 (5.00)	
Sensory deficit in front of thighs	1 (1.01)	0 (0.00)	1 (1.67)	

^#^Chi-squared test

**Table 4:** Clavien–Dindo classification (early and delayed complications)

Post-operative complications	All (N = 99)	BMI < 30 kg/m^2^ (N = 39)	BMI ≥ 30 kg/m^2^ (N = 60)	P value
Early complications				
Grade I	6 (6.06)	2 (5.13)	4 (6.67)	0.7551^#^
Grade II	8 (8.08)	4 (10.26)	4 (6.67)	0.5240^#^
Delayed complications				
Grade I	10 (10.10)	2 (5.13)	8 (13.33)	0.1878^#^

^#^Chi-squared test

Likewise, the Clavien–Dindo classification of complications did not reveal any significant differences among the study groups (Table 4).

### Histopathological findings and post-operative adjuvant treatment

Details regarding histopathological findings and post-operative adjuvant treatment is presented in Table 5.

**Table 5:** Histopathological findings and post-operative adjuvant therapy

Histopathological findings and post-operative adjuvant therapy	All (N = 99)	BMI < 30 kg/m^2^ (N=39)	BMI ≥ 30 kg/m^2^ (N = 60)	P value
Uterine size, mean ± SD, cm	9.03 ± 2.03	8.72 ± 1.66	9.22 ± 2.21	0.2300*
Stage, n (%)				
IA	71 (71.72)	28 (71.79)	43 (71.67)	0.5608^#^
IB	20 (20.20)	7 (17.95)	13 (21.67)	
II	4 (4.04)	1 (2.56)	3 (5.00)	
IIIA	1 (1.01)	1 (2.56)	0 (0.00)	
IIIC	3 (3.03)	2 (5.13)	1 (1.67)	
Type, n (%)				
Endometroid adenocarcinoma	80 (80.81)	31 (79.49)	49 (81.67)	0.9179^#^
Serous carcinoma	10 (10.10)	5 (12.82)	5 (8.33)	
Mixed carcinoma	3 (3.03)	1 (2.56)	2 (3.33)	
Carcinosarcoma	2 (2.02)	1 (2.56)	1 (1.67)	
Clear cell adenocarcinoma	2 (2.02)	1 (2.56)	1 (1.67)	
Mucinous carcinoma	1 (1.01)	0 (0.00)	1 (1.67)	
Small cell adenocarcinoma	1 (1.01)	0 (0.00)	1 (1.67)	
Number of nodes, mean ± SD	23.24 ± 12.00	22.49 ± 11.65	23.76 ± 12.21	0.6079^#^
Radiation therapy, n (%)	47 (47.47)	18 (46.15)	29 (48.33)	0.9776^#^
Chemotherapy, n (%)	8 (8.08)	3 (7.69)	5 (8.33)	0.9430^#^
Hormonal therapy, n (%)	35 (35.35)	16 (41.03)	19 (31.67)	0.6298^#^
Recurrence, n (%)				
Yes	4 (4.04)	2 (5.13)	2 (3.33)	0.6593^#^
No	95 (95.96)	37 (94.87)	58 (96.67)	
Site of recurrence, n (%)				
Abdomen	2 (2.02)	1 (2.56)	1 (1.67)	0.6593^#^
Abdomen, nodal, inguinal, brain	1 (1.01)	1 (2.56)	0 (0.00)	
Abdomen, omentum, muscle abdomen	1 (1.01)	0 (0.00)	1 (1.67)	
Status				
Alive	97 (97.98)	38 (97.44)	59 (98.33)	0.7577^#^
Dead	2 (2.02)	1 (2.56)	1 (1.67)	

*Student’s t-test; ^#^Chi-squared test

Although the patients in the BMI group ≥ 30 kg/m^2^ had a larger mean uterine size (9.22 ± 2.21 vs. 0.2300 cm, P = 0.2300) than those in the BMI group < 30 kg/m^2^, the difference was not statistically significant. Similarly, a higher but statistically insignificant number of nodes were harvested in the BMI group ≥ 30 kg/m^2^ in comparison with the BMI group < 30 kg/m^2^ (23.76 ± 12.21 vs. 22.49 ± 11.65, P = 0.6079).

### DFS and OS

**Fig. 1:**
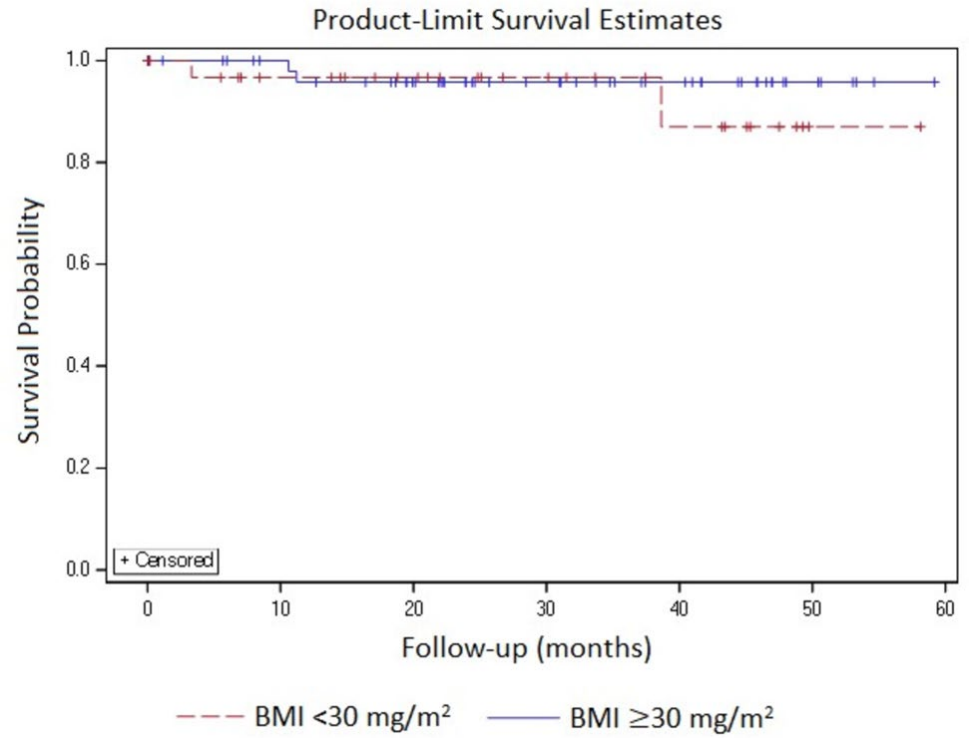
Disease-free survival

**Fig. 2:**
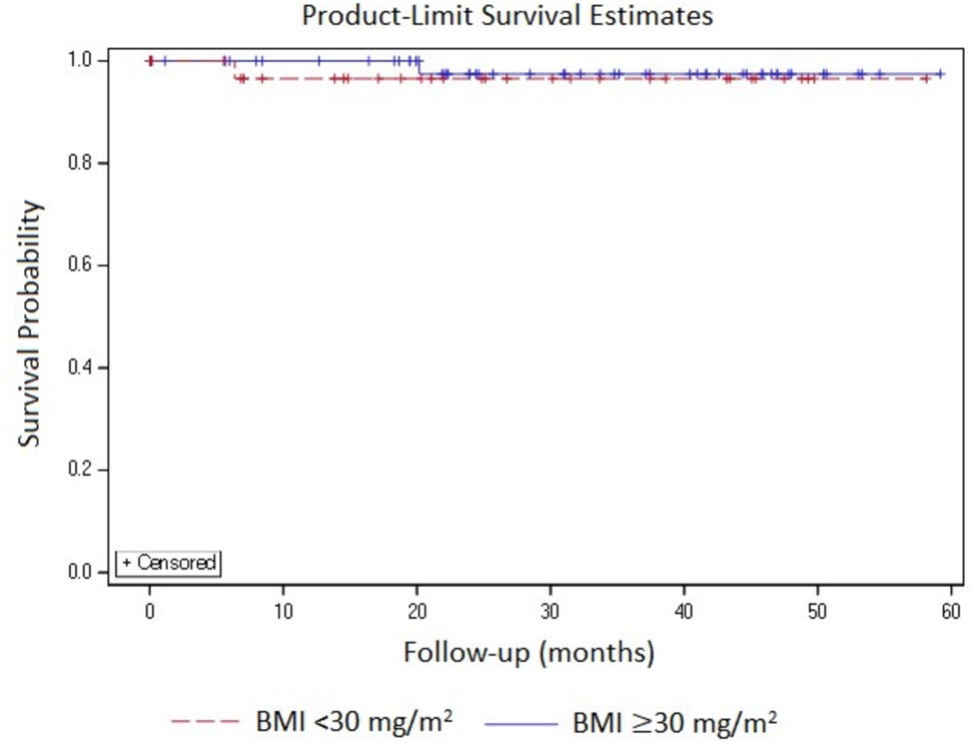
Overall survival

At a median follow-up of 22.3 months (range 0.03–59.17 months), only 4 recurrences (2 in each group) and 2 deaths (1 in each group) were reported. The number of recurrences (5.13% vs 3.33%) and deaths (2.56% vs 1.67%) were more in BMI < 30 kg/m^2^ group. For the length of follow-up, no significant differences were observed in DFS (94.8% and 96.7%) and OS (97.4% and 98.3%) across the study groups. Due to the lesser number of events, the median DFS and OS were not estimable for the study groups (Figs. 1 and 2).

## Discussion

The increase in obesity increases the risk of EC and consequently surgical morbidity regardless of the surgical method used. Nevertheless, the minimal invasive surgery (MIS) approach has significantly reduced the risk compared to the open approach [15]. In 2017, the National Comprehensive Cancer Network (NCCN) guidelines, has recommended hysterectomy via MIS approach for uterine-confined cases [16]. In a retrospective cohort study, four high-volume NCCN institutions reported at least 80% minimally invasive hysterectomy surgery and lower perioperative complications in patients with a mean BMI of 33.7 ± 9.6 kg/m^2^ [9]. A randomized controlled trial (RCT) demonstrated non-inferiority with respect to paraaortic lymph node yield, complication rates, hospital stay, and total cost favoring robotic surgery over the open technique [17]. Another RCT showed that robotic surgery was faster without conversion into open surgery than conventional laparoscopy. However, the surgical outcomes remained similar in both groups [18]. The use of MIS in the management of EC is evolving rapidly in India, but the role of robotic surgery in disease management is still less defined. Given that the ‘overweight-obesity pandemic’ is expected to grow in Asia in the coming years, we wanted to evaluate the surgical outcomes of robotic-assisted technology in Indian EC patients.

A total of 99 patients were included in our study with 39 and 60 patients respectively in BMI groups < 30 and ≥ 30 kg/m^2^. In this latter group, 13 patients were morbidly obese with a BMI of ≥ 40 kg/m^2^. To our knowledge, our study is the largest set of Indian cases that takes these BMI subgroups into account. Two previous retrospective studies from India reported the results of robotic-assisted technology with an average BMI of < 30 kg/m^2^ [19, 20] while one study reported results with an average BMI of 32.39 ± 6.9 kg/m^2^ [21]. Another prospective study included 25 patients with a BMI of 30.96 kg/m^2^ in the robotic arm [13]. Therefore, our extensive and selective assessment of robotic-assisted technology in the obese Indian population, particularly morbidly obese, is unprecedented in the literature. Unlike the previous report [12], more previous surgeries were reported in the BMI group > 30 kg/m^2^ of our study, even though the difference was not statistically significant (P = 0.6038). The major co-morbidities of the two subgroups in our study were hypertension, diabetes, and thyroid disorders (P = 0.4444).

The mean blood loss in our study was 14.85 ± 13.15 ml which was much lower than the two previously published reports from India [8, 13]. While the average blood loss reported in one study was 149.99 ± 85.77 ml in patients with a BMI of 32.39 ± 6.9 kg/m^2^ the other reported a 81.28 ml blood loss in patients with a BMI of 30.96 kg/m^2^. The study by Geppert et al. reported a median blood loss of 100 ml (range 0–400 ml) and 50 ml (range 25–200 ml) in obese (BMI = 30–34.9 kg/m^2^) and morbidly obese women (BMI ≥ 35 kg/m^2^) undergoing robot‐assisted laparoscopic hysterectomy [22]. Another study by King et al. [23] reported an increase in blood loss (72.2 vs. 66.4 ml, P = 0.2) along with increased BMI (33.2 vs. 37.5 kg/m^2^). Similarly, another study by Lau et al. reported significantly higher blood loss (64.1 vs. 95.9 ml, P < 0.05) in patients with BMI values of 34.2 kg/m^2^ than 25.2 kg/m^2^ [12]. Interestingly, no significant difference in blood loss (12.76 ± 8.64 vs. 16.19 ± 15.22 ml, P = 0.2041) was seen in our subgroups. In other words, blood loss was significantly reduced during robotic surgery regardless of the patient’s BMI. In addition, 10.6% of patients required a blood transfusion in a previous study from India [21] while no blood transfusion was required in this study.

Few authors suggest that robotic-assisted surgery is more time-consuming than the open method [13]. The mean operating time observed in this study was 282.77 ± 69.95 min, slightly higher than the previous studies [8, 24]. The total operating time (284.36 ± 80.85) in our study was slightly higher in the BMI group < 30 kg/m^2^ as the subordinate staff was given the learning opportunity during the surgeries of low-risk, non-obese patients. However, the operative time of our study was not affected by the BMI of the subgroups (p = 0.8552). This observation was consistent with the observations reported in earlier published papers [11, 12]. Our results were also consistent with an earlier study in which operating time was higher in women with a BMI of ≥ 30 kg/m^2^ than in women with a BMI of < 30 kg/m^2^ (255 ± 64 min vs. 237 ± 54, min, P = 0.13) without a statistically significant difference [12].

The length of hospital stay in this study was 1.62 ± 0.49 days for the BMI group < 30 kg/m^2^ and 1.68 ± 0.74 days for the BMI group ≥ 30 kg/m^2^ (P = 0.6564). Hospital stay of less than 2 days in both subgroups of our study corroborate the results of many previous studies [21, 25]. Nevertheless, one study also found a higher median length of stay of 3.11 ± 0.89 days with a BMI of 32.39 ± 6.9 kg/m^2^ [21]. In addition, in one study, a prolonged 44 days of hospitalization in an obese patient was observed [3]. None of these aberrant cases were observed in our study.

With the advent of robotic surgery, fewer complications are observed, especially in the obese and high-risk population [26]. Although few studies have identified both intra and post-operative complications in obese women [12, 15], the number of complications between our subgroups, although higher in the BMI group ≥ 30 kg/m^2^, has achieved no statistical significance. A similar finding was published by Kannisto et al. with no difference in the number of complications among individuals with BMI < 30 and ≥ 30 kg/m^2^ [24]. Another study showed that the relative risk ratio of grade II–V complications by Clavien–Dindo classification, was 0.54 (95% CI 0.31–0.93) for the robotic group as compared to the open group in patients with BMI > 30 kg/m^2^ [15]. However, the Clavien–Dindo classification of the complications in our study showed no significant differences between the study groups.

In addition, our study also included 13 morbidly obese patients with a BMI of ≥ 40 kg/m^2^. The blood loss, operating time, and hospital stay in this group were 18.85 ml, 266.62 min, and 1.69 days respectively, as in the other subgroups. Furthermore, no complications have been revealed in morbidly obese women. We consider this a remarkable finding compared with previous reports which showed significantly higher blood loss of 94 ml (BMI = 45.8 kg/m^2^) [12], 68.0 ml (BMI > 43.5 kg/m^2^), 96.7 ml (BMI > 52.6 kg/m^2^) [23] and 85 ml (BMI > 40 kg/m^2^) [11] respectively. The length of hospital stays has also increased with the increased BMI, particularly in morbid and extremely morbid patients (BMI > 50 kg/m^2^). With the added advantage of shorter/similar hospital stays, robotic technique has shown a clear advantage in the obese and morbidly obese population in our hospital setting. We agree with the common belief that a higher surface area of obese or morbidly obese people can facilitate the proper placement of the port and the easy movement of the arms leading to better results [13].

In our study, the two subgroups were comparable, with no statistical differences in uterine size, stage, type, and in the number of patients receiving adjuvant treatment. Although lymph node yield was higher in the BMI group ≥ 30 kg/m^2^, the difference was not statistically significant (22.49 ± 11.65 vs. 23.76 ± 12.21, P = 0.6079). The number of nodes retrieved by our group was higher than in the previous studies [15, 23, 25] perhaps because of the ease of lymphadenectomy by robots. In contrast to our study, higher lymph node retrieval has been reported in a few previous studies [8, 11].

LAP2 study by Gunderson et al. [27] showed that obese people had a higher risk of overall death irrespective of the surgical technique, but a similar risk of disease-specific death, compared to non-obese people. At a median follow-up of 22.3 months, there were 2 recurrences and 1 death in each of the two subgroups. Although for a shorter follow-up period, the median OS of 98.3% in the BMI group ≥ 30 kg/m^2^ of our study was higher than the 5-year OS of 87% reported by one study in women with a median BMI of 36 kg/m^2^ [15]. Similarly, the DFS reported in the same study [15] was 81.6% versus 96.7% in our ≥ 30 kg/m^2^ BMI subgroup. The study also reported 13 recurrences and 11 deaths in the robotic arm [15]. Another Indian study reported 5-year DFS and an OS of 89.68% and 89.25% respectively in the robotic surgery group [21]. Similarly, a 3-year DFS of 83.3% was also reported in the past [28]. Hinshaw et al. showed better OS and DFS in all patients with ≥ 35 kg/m^2^ BMI than the open technique (P > 0.05) which corroborates our results [29]. In accordance with our findings, a study by Safdieh et al. also reported an OS of 96.1% after a 25 months median follow-up [25]. Consequently, our result so far indicates desirable OS and DFS in the obese population and thus, reinforces the use of robotic technology.

While previous reports have shown a higher blood loss, operating time, hospital stay, and complications with increased BMI [23], our study found similar surgical outcomes in non-obese and obese women with a notable benefit in morbidly obese women. This may be explained by the fact that the learning curve of the primary surgeon was achieved a long time ago. The ergonomics of the robotic technique eliminates the pivotal effect and the typical poor visualization of laparoscopy in the obese population [30] and thus avoids conversion into open surgery. Moreover, the ease of spatial movement, without tremors, of the robotic hands allows surgeons to easily access difficult anatomies. Therefore, we suggest that robots can be used successfully in all obese subgroups undergoing robotic-assisted surgery for EC.

### Strengths and limitations

To our knowledge, this study is the first to evaluate the success of robotic-assisted technology in India by considering the BMI of the existing population. A relatively small number of patients within each group as well as the retrospective design may be considered a limitation. Furthermore, due to the shorter median follow-up time, survival outcomes related to DFS and OS could not be estimated. However, similar surgical outcomes, independent of the BMI of the study population, clearly outweigh the limitations of the study.

### Future perspectives

With more experience and accessibility to robots, the increasingly older and obese population would be successfully treated with fewer complications. A prospective study assessing long-term outcomes like DFS and OS after robotic-assisted surgery in Indian EC patients seems justified.

## Conclusion

A causal association between the increase in BMI and the risk of EC is well documented. Despite this, only a few studies have attempted to assess the benefits of robotic-assisted technology among obese versus non-obese population. As obese patients can present greater treatment challenges related to size, comorbidities, postoperative outcomes, etc., this subgroup is often excluded from clinical trials as well. Given this, our study suggests that elderly and obese women predisposed to multiple medical co-morbidities and surgical complications would especially benefit from robotic-assisted technology regardless of their BMI. Even though the same is criticized for its higher acquisition cost (20–40% more than the open surgery) the reduced use of analgesics, antiemetics, IV fluids, and blood transfusion can clearly offset the total cost of the treatment. In addition, many hospitals now consider robots to be a one-time investment and are therefore inclined to have at least one robotic system available for their facility.

## Data Availability

All data supporting the findings of this study are available within the paper.
